# Exploring Taiwanese Consumer Dietary Preferences for Various Vinegar Condiments: Novel Dietary Patterns across Diverse Cultural Contexts

**DOI:** 10.3390/nu15173845

**Published:** 2023-09-03

**Authors:** Jung-Kuei Ker, Ching-Sung Lee, Yen-Cheng Chen, Ming-Chen Chiang

**Affiliations:** 1 Ph. D. Program in Nutrition and Food Science, College of Human Ecology, Fu Jen Catholic University, New Taipei City 242062, Taiwan; 2 Department of Food Beverage Management, Mackay Junior College of Medicine, Nursing and Management, Taipei 11260, Taiwan; 3 Department of Restaurant, Hotel and Institutional Management, College of Human Ecology, Fu Jen Catholic University, New Taipei City 242062, Taiwan; 4 Department of Applied Science of Living, College of Agriculture, Chinese Culture University, Taipei 11114, Taiwan

**Keywords:** food choice, black vinegar, balsamic vinegar, sensory evaluation, food psychology, consumer acceptance, innovation acceptance, Taiwan

## Abstract

The use of vinegar as a culinary seasoning in various global cuisines to enhance the taste characteristics and profiles of foods has been extensively documented in the culinary literature. Particularly notable is traditional Taiwanese-style thick soup, where the incorporation of vinegar plays a fundamental role in imparting distinct flavors. In the context of this experimental investigation, the foundational base of Taiwanese-style thick soup serves as the platform for a meticulously planned sensory and dietary behavior evaluation. Our research methodology combines the use of survey questionnaires and experimental techniques, employing purposive sampling and snowball sampling methods to recruit participants. The central focus of this study is to understand consumers’ culinary preferences when presented with a choice between two contrasting types of vinegar—specifically, black vinegar and balsamic vinegar—as alternative gastronomic enhancements. This precise orchestration of data collection and systematic evaluation provides a perceptive window into participants’ culinary inclinations and food choices, resulting in a detailed and profound understanding of their taste preferences. The empirical findings stemming from this experimentation reveal notably significant differences in the sensory assessments among participants engaging in diverse culinary experiences. Notably, distinct variations are observed in terms of visual perceptions, olfactory distinctions, and overall sensory satisfaction. This study occupies a crucial position within existing research paradigms by strategically expanding the scope of sensory investigations within the realm of Taiwanese-style thick soup. This introduces an innovative aspect represented by the introduction of balsamic vinegar as a compelling alternative to the customary black vinegar. As a result, the emerging findings not only offer compelling insights into the nuanced food choice and taste preferences of consumers, but also open up new and innovative directions within the complex tapestry of Chinese gastronomy.

## 1. Introduction

In Taiwanese food culture, soups are an important part of daily diet. Besides being tasty and nutritious, drinking soup can also provide health benefits to the human body, and diversified soups can make our lives better [1]. In particular, the addition of vinegar to soup can affect its taste [2]. Incorporating condiments alters the sensory attributes of food, including its color, texture, and aroma, thereby producing different sensory effects [3]. Soup recipes and ingredient compositions vary across different regions, influenced by culinary cultures and the availability of seasoning components, resulting in a wide array of unique culinary creations. Despite the numerous factors contributing to soup variations, most soups share common key sensory attributes and are regarded as favored food choices by consumers [4].

Vinegar has long been recognized in many Asian countries as an important culinary ingredient [5,6], either as a condiment for food or as an ingredient in a dish, as it may alter the overall preference for a given food or meal [7]. Notably, Chinese-style cuisine is renowned for its dense and exquisitely flavored dishes [8]. In Taiwan, the addition of black vinegar (a variant of grain vinegar) to soups is very popular, bringing about a distinctive black hue [9]. The common black vinegar in Taiwan is a traditional rice vinegar [10], mostly used as a condiment with rice, wheat, millet, sorghum, or a combination of these, and its color tends to be amber. When incorporated into a dish, it brings about a subtle sourness that immediately transforms the taste into a delicious and inviting sensation, often referred to as “black vinegar”. Taiwanese-style thick soup is made by boiling shredded mushrooms, shredded Chinese cabbage, and shredded bamboo shoots, and finally thickened with starchy water, adding suitable ingredients, seasonings, and black vinegar to present a thick soup with rich flavor layers that are loved by consumers, making it a famous home-cooked dish in Taiwan.

Conversely, in European countries, high-quality balsamic vinegar is preferred by numerous individuals and is extensively utilized across various types of food. The term “balsamic vinegar” is commonly employed to designate sauces, dressings, and condiments with a distinct sweetness [11]. Balsamic vinegar enhances food applications by introducing unique flavors, particularly when incorporated into culinary preparations, thereby creating a distinctive gustatory experience. The popularity of balsamic vinegar (wine vinegar) as a seasoning has witnessed an upward trend among the general populace [12].

Previous research has primarily focused on evaluating the sensory perception, encompassing taste, aroma, and overall acceptance of diverse food and beverage items to discern consumer preferences [13]. However, it is crucial to recognize that sensory science plays a vital role in the product development process preceding commercial production, as it serves as a crucial link between producers and consumers, with human sensory perception being of the utmost importance. Hence, investing in sensory evaluation is imperative [14]. This approach allows researchers to gather valuable information about product attributes and overall preferences, thereby enhancing the overall quality characteristics [15]. As a result, a wide range of scales have been developed to quantify the levels of preference for such products [7,16]. Extensive investigations have been conducted to examine the preference and satisfaction levels regarding different types of vinegar, considering various sensory characteristics. For example, Short, Kinchla, and Nolden [14] conducted a sensory evaluation study to understand how to increase consumer preference for plant-based cheese substitutes; Lee et al. [17] conducted a study on the effect of eliciting context on consumer expectations and its effect on their subsequent satisfaction and sensory evaluation; Craine et al. [18] used consumer sensory evaluations of malt and beer to understand the acceptance of beer in terms of aroma, appearance, taste/flavor, sweetness, and overall preference; and Grasso et al. [19] studied the effect of consumers’ sensory evaluation of three types of burgers, answering questions about preference, check-all-that-apply, willingness to buy, and willingness to pay in a blinded experiment.

Moreover, consumers exhibit discerning behavior when evaluating sensory attributes such as color, texture, and flavor in food, highlighting the pivotal role of determining the overall acceptability and sensory properties of food products within the food industry [20]. In the context of the taste evaluation of food products, two primary factors assume prominence: consumers’ perception of product distinctions and their comprehensive evaluations or preferences for each product [21]. Sensory evaluation methods can provide a deeper understanding between the perception of sensory attributes and their effects on liking/disliking [14]. Not only can consumers sometimes detect lower levels of odor and other sensory attributes than instruments, but instruments cannot measure pleasure or predict preferences in the same way that humans can [22]. Thus, Reinaa et al. [23] proposed the adoption of sensory evaluation methods to assess diverse vinegar types and their impact on preferences across a wide range of food and beverage items.

Consumers tend to reject or avoid unfamiliar foods [24]. In order to comprehensively understand the acceptance and purchase intentions of innovative foods in the market [25], it is necessary to consider conducting experimental designs for innovative product development. This enables an understanding of consumer preferences for novel seasonings, facilitating the creation of products that better align with consumer expectations. The identification of consumer segment preferences is crucial for food manufacturers to strategically position innovative products in the market and formulate effective strategies.

Building upon previous research, the main objective of this study is to conduct sensory evaluations and explore consumer food preferences by incorporating black vinegar and balsamic vinegar into Taiwanese-style thick soup. The sensory evaluation includes visual, olfactory, taste, and overall perceptions, capturing participants’ viewpoints. Through the adoption of this methodology, our goal is to gain a comprehensive understanding of consumers’ sensory preferences and the extent of their dietary choices. Ultimately, we expect that the experimental design and findings of this research will contribute to the innovative utilization of Chinese cuisine.

## 2. Materials and Methods

### 2.1. Participants

This study enrolled 200 participants through purposive and snowball sampling methods, all of whom were affiliated with Mackay College of Nursing and Management & Fu Jen Catholic University in Taipei, Taiwan. Among these participants, 11 were excluded from the data analysis due to incomplete engagement, resulting in a final sample size of 189 individuals. The demographic characteristics of the participants are detailed in Table 1. All the participants self-reported consuming Taiwanese-style thick soup with a frequency of at least once every 2–3 months. They also confirmed being free from any significant illnesses or conditions that might potentially affect their taste or sense of smell, thus affirming their physiological well-being.

The participants were specifically chosen based on their use of vinegar as a sensory seasoning ingredient in Taiwanese-style thick soup. They were requested to provide information about their preferred flavors and the establishments where they typically bought Taiwanese-style thick soup. After completing the sensory evaluation of the samples, the participants were compensated with gift cards as a gesture of gratitude for their involvement.

### 2.2. Experimental Materials

In this experiment, Taiwanese thick soup was chosen as the primary base, incorporating ingredients such as eggs, shredded bamboo shoots, and coriander. This specific soup base is frequently encountered in various dining establishments throughout Taiwan. Traditionally, the addition of meat or seafood is customary in this soup; however, for the purpose of this study, a deliberate decision was made to exclude such ingredients. This was done to ensure that the introduction of meat or seafood would not compromise the primary flavor characteristics of the vinegar component within the soup (Table 2).

In the sensory evaluation analysis, the participants were presented with 240 milliliters of Taiwanese-style thick soup, to which 5 milliliters of either black vinegar or balsamic vinegar was added for sensory evaluation purposes (which is typically the standard serving size for such Taiwanese-style soups in Taiwan). It is important to note that the sensory characteristics of most foods can only be accurately evaluated by participants through well-executed, comprehensive, and meaningful assessments, rather than relying solely on scientific instruments. However, it is equally crucial to ensure that participants’ behavior closely emulates that of scientific instruments as much as possible. To achieve this, the strict control of all testing methods and conditions becomes necessary in order to mitigate the potential influence of psychological factors on the measurement outcomes [26].

In this experiment, the control group consisted of the traditional Taiwanese-style thick soup, commonly seasoned with black vinegar, while the experimental group involved the innovative Taiwanese-style thick soup incorporating balsamic vinegar, which is not typically used in Chinese cuisine. The black vinegar used in the experiment is produced from rice, while the balsamic vinegar is produced from grapes. Both types of vinegar are commercial products, and black vinegar is the most common brand used in Taiwan. In Taiwan, black vinegar is made by stewing vegetable juice, waiting for the onion, celery, garlic, and carrot, etc. to boil out and then draining it off, adding the broken spices, and finally adding sugar, salt, vinegar, and sauce to make it. To minimize the potential interference from different ingredients, only the liquid portion of the soup was sampled for the participants to taste. Furthermore, the participants were deliberately not informed about the type of vinegar added to the soup in order to prevent any preconceived bias that could potentially influence the sensory evaluation results.

### 2.3. Procedure

To examine the acceptance of innovative Taiwanese-style thick soup when traditional Taiwanese-style thick soup is subjected to seasoning innovation, we designed a sensory evaluation experiment comparing traditional Taiwanese-style thick soup with innovative Taiwanese-style thick soup. Through a sensory evaluation incorporating visual, olfactory, gustatory, and overall assessments, we collected the participants’ opinions. We aim to provide new innovative developments to the market of traditional Chinese cuisine through the experimental results obtained from this research.

This research protocol obtained approval from the Institutional Review Board of Mackay Junior College of Medicine, Nursing, and Management (Approval Number: MKC112R12). Prior to their participation, each participant was thoroughly briefed on the experimental procedures and provided written informed consent. The participants were instructed to refrain from smoking, eating, or drinking any fluids (except water) for at least 2 h before entering the experimental site for their sample evaluation [27]. The experimental site for sample evaluation was prepared with an air purifier to eliminate any odors in the air that could affect olfactory perception. Additionally, the evaluation took place in a brightly lit environment with white lighting to avoid any color-related influences on visual perception. Furthermore, the experimental environment was ensured to be free from any external noise, creating a quiet and safe experimental setting where each participant underwent sample evaluation individually.

**Figure 1:**
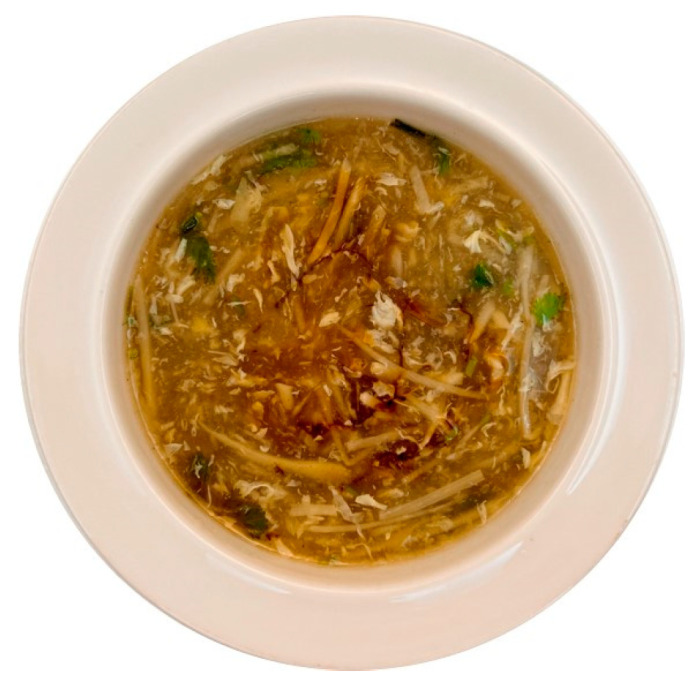
Traditional Taiwanese-style thick soup.

**Figure 2:**
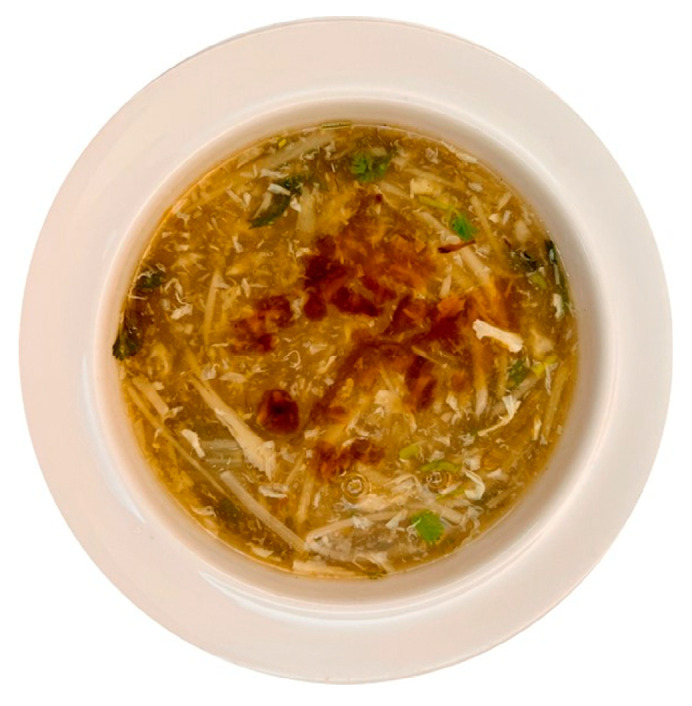
Innovative Taiwanese-style thick soup.

Prior to the sample evaluation, the participants were asked to independently consume small amounts of both black vinegar and balsamic vinegar as the grouping criteria for preference between traditional and innovative condiments. The participants were unaware of their assigned groups. Once the grouping was completed, the researchers prepared traditional Taiwanese-style thick soup (Figure 1) and innovative Taiwanese-style thick soup (Figure 2) in white porcelain bowls, ensuring the soup temperature was at 60 °C [2]. To prevent the participants from being influenced by the flavors of different samples during the experiment, they were instructed to rinse their mouths after tasting each sample. To avoid using colder water that could affect their sensory perception when rinsing their mouth, colorless and tasteless warm water (40 ± 1 °C) was provided for mouth rinsing, eliminating any residual taste and serving as a mouth cleanser before completing the sensory evaluation questionnaire. Subsequently, the participants proceeded to the next sample, following a predetermined order without any arbitrary changes, to maintain the highest level of authenticity and rigor in the experimental procedure.

Based on the research findings of Swiader and Marczewska [28] regarding sensory evaluation factors in terms of visual, olfactory, gustatory, and overall flavor aspects, the following definitions were formulated for the sensory evaluation factors:

(1) Visual appearance: This assessment primarily focuses on the participants’ preference for the visual presentation of Taiwanese-style thick soup when it is prepared in glass containers. It also explores their preference regarding the integration of color by combining the soup with an equal amount of either black vinegar or balsamic vinegar.
(2) Olfactory aroma: This refers to the participants’ perception of the aroma when smelling the samples. The black vinegar used in the experiment is produced from rice, while the balsamic vinegar is produced from grapes.
(3) Gustatory taste: This evaluation centers on the participants’ perception of the taste characteristics in Taiwanese-style thick soup, encompassing sourness, sweetness, bitterness, spiciness, saltiness, and umami.
(4) Overall flavor: This pertains to the participants’ preference for the overall flavor of the samples.

The questionnaire primarily comprised items pertaining to sensory evaluation. This instrument was formulated through a pilot study conducted by a panel of experts. The composition of the panel encompassed three professors from university departments specializing in food and nutrition, along with three industry experts from vinegar-manufacturing establishments. Rigorous expert calibration was conducted to validate the questionnaire, and its reliability was ascertained through a reliability test, yielding a favorable Cronbach’s α coefficient of 0.89. The items within the questionnaire encompassed aspects such as visual appearance, olfactory aroma, gustatory taste, and overall evaluation (as presented in Table 3).

The participants were tasked with completing the sensory evaluation questionnaire utilizing a Likert scale featuring five gradations. This questionnaire was administered after the participants’ consumption of Taiwanese-style thick soup paired with traditional condiments (control group) and the innovative iteration of Taiwanese-style thick soup accompanied by innovative condiments (experimental group).

### 2.4. Data Analysis

The statistical analysis software SPSS 24.0 for Windows was used for the data analysis. Firstly, the participants’ preferences for vinegars were grouped, and then the data related to the sensory evaluation were analyzed, including narrative statistics and a mean-checking analysis.

## 3. Results

### 3.1. Independent Samples T-Test

The Taiwanese-style thick soup was divided into traditional Taiwanese-style thick soup (with the addition of black vinegar) and innovative Taiwanese-style thick soup (with the addition of balsamic vinegar). Traditionally, the primary condiment used in Taiwanese-style thick soup is black vinegar [9]. Thus, the concepts of traditional and innovative condiments were adopted as the foundation for this study. This approach aimed to ensure the homogeneity of the participants’ perceptions of the condiments. Through the experiment, the study sought to explore the innovation of condiments in Taiwanese-style thick soup and examine whether the participants would perceive and accept it differently. Therefore, homogeneity of variance tests and independent samples t-tests were conducted. After the grouping analysis, as shown in Table 4, no significant differences were found between the traditional and innovative condiment groups (*p* > 0.05). Traditional seasoning (black vinegar) mean(M) = 3.27, Standard Deviation(SD) = 0.76, and Innovative seasoning (balsamic vinegar) mean(M) = 3.45, Standard Deviation(SD) = 0.81. This showed that the subjects preferred the innovative seasoning (balsamic vinegar).

### 3.2. Analysis of Visual Sensory Evaluation of Traditional Taiwanese-Style Thick Soup and Innovative Taiwanese-Style Thick Soup

Regarding the visual sensory evaluation of the traditional Taiwanese-style thick soup and innovative Taiwanese-style thick soup, the results of the independent samples t-test analysis showed significant differences in the visual sensory perception of the traditional Taiwanese-style thick soup (*t* = −2.98, *p* < 0.01) and innovative Taiwanese-style thick soup (*t* = −2.31, *p* < 0.05), as presented in Table 5. In the control group, M = 2.53, SD = 0.83 for the traditional dressing (balsamic vinegar) and M = 3.71, SD = 0.73 for the innovative dressing (balsamic vinegar); and in the experimental group, M = 2. 93, SD = 0.93 for the traditional dressing (balsamic vinegar) and M = 4.00, SD = 0.86 for the innovative dressing (balsamic vinegar). Thus, it can be seen that both groups had a higher sensory visual preference for the innovative seasoning (balsamic vinegar).

### 3.3. Analysis of Olfactory Sensory Evaluation of Traditional Taiwanese-Style Thick Soup and Innovative Taiwanese-Style Thick Soup

Regarding the olfactory sensory evaluation of the traditional Taiwanese-style thick soup and innovative Taiwanese-style thick soup, the results of the independent samples t-test analysis revealed significant differences in the olfactory sensory perception of the traditional Taiwanese-style thick soup (*t* = −2.49, *p* < 0.05) and innovative Taiwanese-style thick soup (*t* = −3.04, *p* < 0.05), as presented in Table 6. In the control group, M = 3.04, SD = 0.91 for the traditional dressing (balsamic vinegar) and M = 4.10, SD = 0.88 for the innovative dressing (balsamic vinegar); and in the experimental group, M = 3.36, SD = 0.75 for the traditional dressing (balsamic vinegar) and M = 4.47, SD = 0.70 for the innovative dressing (balsamic vinegar). Thus, it can be seen that both groups had a higher sensory olfactory preference for the innovative seasoning (balsamic vinegar).

### 3.4. Analysis of Taste Sensory Evaluation of Traditional Taiwanese-Style Thick Soup and Innovative Taiwanese-Style Thick Soup

An average mean comparison analysis was conducted on the taste sensory evaluation of the traditional Taiwanese-style thick soup and innovative Taiwanese-style thick soup. The results indicated non-significant differences in the taste sensory evaluation for the traditional Taiwanese-style thick soup (*t* = −1.81, *p* > 0.05) and innovative Taiwanese-style thick soup (*t* = −1.71, *p* > 0.05), as presented in Table 7. In the control group, M = 3.34, SD = 1.06 for the traditional dressing (balsamic vinegar) and M = 4.09, SD = 0.75 for the innovative dressing (balsamic vinegar); and in the experimental group, M = 3.60, SD = 0.80 for the traditional dressing (balsamic vinegar) and M = 4.28, SD = 0.68 for the innovative dressing (balsamic vinegar). Thus, it can be seen that both groups had a higher sensory taste preference for the innovative seasoning (balsamic vinegar).

### 3.5. Analysis of Overall Sensory Evaluation of Traditional Taiwanese-Style Thick Soup and Innovative Taiwanese-Style Thick Soup

The results of the average mean comparison analysis revealed significant differences in the overall sensory evaluation between the traditional Taiwanese-style thick soup and innovative Taiwanese-style thick soup. The traditional Taiwanese-style thick soup demonstrated significant differences in its overall sensory evaluation (*t* = −3.12, *p* < 0.01), while the innovative Taiwanese-style thick soup also showed significant differences in its overall sensory evaluation (*t* = −2.40, *p* < 0.05), as shown in Table 8. In the control group, M = 3.10, SD = 0.88 for the traditional dressing (balsamic vinegar) and M = 3.88, SD=0.88 for the innovative dressing (balsamic vinegar); and in the experimental group, M = 3.54, SD = 0.94 for the traditional dressing (balsamic vinegar) and M = 4.20, SD = 0.85 for the innovative dressing (balsamic vinegar). Thus, it can be seen that both groups had a higher sensory overall preference for the innovative seasoning (balsamic vinegar).

**Figure 3:**
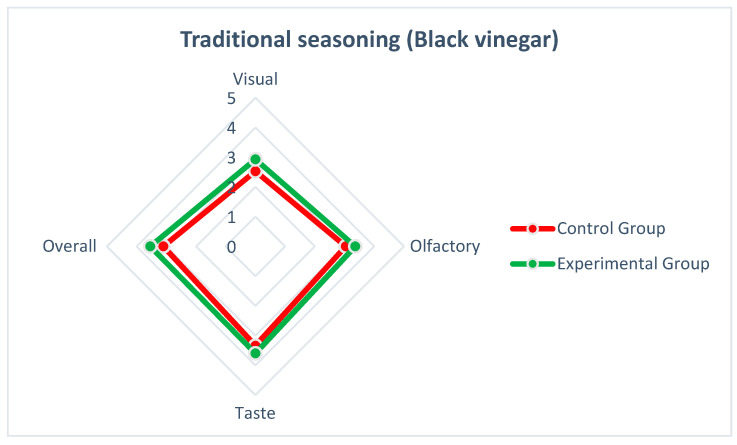
Sensory evaluation radar chart of Traditional seasoning (Black vinegar).

**Figure 4:**
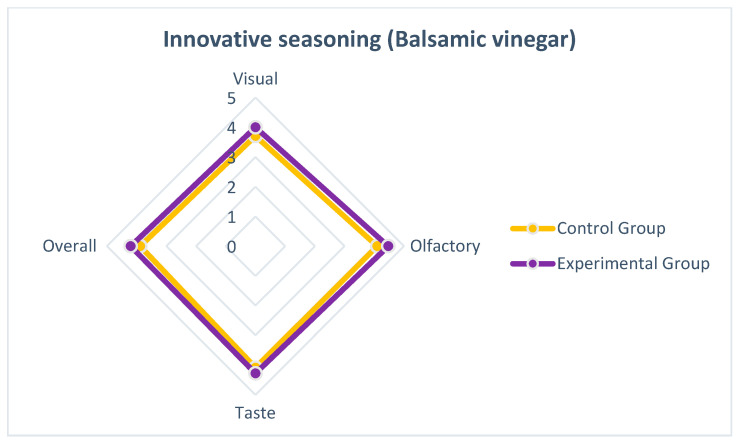
Sensory evaluation radar chart of Innovative seasoning (Balsamic vinegar).

We posit that individuals’ sensory evaluations of a particular food in their dietary culture are heavily influenced by long-term exposure [11,29]. This holistic, subjective sensory perception is shaped by the visual, olfactory, and gustatory aspects associated with the prolonged consumption of the food [30]. As a result, when enjoying this food, individuals tend to add their preferred condiments, influenced by factors such as lifestyle, culture, and habits [31]. In our experiment, the participants were divided into groups based on traditional condiments (black vinegar) and innovative condiments (balsamic vinegar), and a sample evaluation was conducted. The research results consistently revealed that the preference for traditional Taiwanese-style thick soup was influenced by participants’ familiarity with their dietary culture [32]. However, the data analysis also showed a favorable response from the participants towards the innovative Taiwanese-style thick soup (Figure 3 and Figure 4). In terms of visual, olfactory, gustatory, and overall sensory perception, the differences between the innovative condiment (balsamic vinegar) and traditional condiment (black vinegar) were minimal. Particularly, in terms of visual perception, the innovative condiment (balsamic vinegar) was perceived more favorably than the traditional condiment (black vinegar). Hence, the research findings contribute to the sensory evaluation of Taiwanese-style thick soup by exploring the replacement of the traditional condiment (black vinegar) with an innovative condiment (balsamic vinegar), filling a gap in this area of study.

## 4. Discussion

### 4.1. Sensory Evaluation of Visual Perception

The findings of this study revealed that the participants rated the innovative Taiwanese-style thick soup higher than the traditional Taiwanese-style thick soup in terms of the visual sensory evaluation. These results are consistent with Tu et al.’s study [33], which suggested that the addition of black vinegar or balsamic vinegar to Taiwanese-style thick soup can elicit different visual perceptions. Therefore, the visual appearance of Taiwanese-style thick soup is important to participants, as it influences their visual perception and expectations for a favorable eating experience. Such perceptions can induce psychological anticipatory effects on consumers and demonstrate that the visual appearance of food can stimulate consumer choices. Treisman’s influential visual perception model [34] proposed that the combination of individual visual features (color and shape) results in a coherent perception, which directs visual attention to these features. From a food perspective, establishing consumer responses to sensory visual attributes such as appearance, texture, and flavor is crucial [35]. However, sensory visual responses to food are modulated by factors including consumer physiology and psychology, as well as learned social and cultural expectations [36]. Therefore, considering the participants’ Chinese dietary culture, where traditional Taiwanese-style thick soup commonly utilizes black vinegar as its added seasoning, the visual perception elicited by the innovative Taiwanese-style thick soup with balsamic vinegar created a more appealing differentiation for the participants.

### 4.2. Sensory Evaluation of Olfactory Perception

The results of this study indicated that the participants rated the traditional Taiwanese-style thick soup higher than the innovative Taiwanese-style thick soup in terms of the olfactory sensory evaluation. The research team believes that the olfactory perception of Taiwanese-style thick soup with the addition of black vinegar is already an established and familiar odor in participants’ dietary culture, with a preference for its aroma [37,38]. Therefore, the choice of a familiar and accustomed flavor was a more expected outcome for the participants, considering that black vinegar and balsamic vinegar differ in their raw materials and the aromas they present. The results of this study confirmed the participants’ olfactory perception of Taiwanese-style thick soup with the addition of black vinegar. Thus, olfactory characteristics play a stimulating role in food consumption and can influence users’ interests and preferences, demonstrating their regulatory function and impact on food intake. The enhancement of food odor and flavor occurs through continuous exposure and contact before and during consumption, influencing the pleasure experienced by a consumer, which aligns with previous studies by Razran [39] and Whitea et al. [40].

### 4.3. Sensory Evaluation of Taste Perception

Based on the research findings, it was observed that the participants consistently rated the traditional Taiwanese-style thick soup higher in the gustatory sensory evaluation when compared to the innovative Taiwanese-style thick soup [41]. The research team posits that this preference for the gustatory perception of the Taiwanese-style thick soup with the addition of black vinegar may be attributed to the participants’ habitual taste memory. In other words, the individuals’ previous experiences with the traditional version of the soup shaped their taste preferences, leading to a preference for its specific flavor profile. Moreover, the study revealed that a combination of different vinegars can have an impact on consumers’ level of liking. This suggests that individuals tend to employ their preferred condiments while consuming food to enhance or elevate its taste, thereby stimulating or satisfying their sensory taste expectations. Over time, as individuals establish a familiar taste preference, they consistently utilize their favored condiments, gradually adapting to this preferred unique taste. This process ultimately contributes to the development of an individual’s own dietary culture and specific taste preferences.

This study’s findings highlight the influence of habitual taste memory and the role of condiments in shaping individuals’ gustatory preferences. Understanding these mechanisms can provide valuable insights into the complex interplay between sensory perception, cultural factors, and individual taste preferences. Further research in this area could delve deeper into the cultural and psychological aspects that contribute to the formation of dietary preferences and potentially offer opportunities for culinary innovation and customization based on individual preferences.

### 4.4. Sensory Evaluation of Overall Perception

Based on the study results, the participants consistently rated the traditional Taiwanese-style thick soup higher than the innovative Taiwanese-style thick soup in terms of the overall sensory evaluation. The research team believes that individuals possess a holistic, subjective understanding of sensory analysis and use cognitive processes to collect sensory analysis data, demonstrating significant differences among the participants due to factors such as culture, education, environment, habits, weaknesses, sensory abilities, and variability in preferences [42]. Black vinegar has long been used as a traditional condiment in Chinese ethnic cuisine, while balsamic vinegar represents a traditional condiment in Italian cuisine. Different ethnic dietary cultures have varying preferences and choices for culinary condiments [3]. These differences in the acceptance of food may depend on the content of ethnic food cues [43] and novelty [44]. In terms of the overall sensory evaluation, black vinegar has become a representative and commonly used condiment, aligning with the concept of Eastern flavor principles [32]. Therefore, although the traditional Taiwanese-style thick soup with black vinegar as a condiment was preferred by the participants, the innovative Taiwanese-style thick soup with balsamic vinegar also garnered a certain level of preference among the participants.

In conclusion, people’s food and beverage preferences are subject to evolving trends, and the fusion of diverse national cultures has given rise to new dietary patterns. This phenomenon is particularly prominent in Taiwan, which is renowned for its cuisine [45]. As consumers increasingly prioritize innovative food and drink options, the integration of different culinary traditions across nations may well become a future trend in production [46]. In the context of consumers’ pursuit of “new experiences” [25,47,48], the culinary landscape is becoming more diverse and inventive, with traditional cuisines undergoing transformation through the innovative application of techniques and ingredients. Against this backdrop, food suppliers should consider the amalgamation of varied ethnic culinary traditions to elevate consumers’ gastronomic experiences and provide them with a more immersive and rewarding culinary journey.

## 5. Conclusions

### 5.1. Conclusion

This study has made a notable contribution to the field by investigating the sensory evaluation of Taiwanese-style thick soup with different condiments. The primary aim was to conduct a comparative analysis between the traditional Taiwanese-style thick soup with black vinegar and an innovative Taiwanese-style thick soup with balsamic vinegar, encompassing various sensory dimensions such as visual perception, olfactory sensations, gustatory experiences, and overall sensory ratings. The obtained results demonstrated statistically significant disparities in the sensory evaluation among the participants who consumed the two distinct soup variations (*p* < 0.05). Consequently, these findings not only facilitate the advancement of academic knowledge within the field, but also emphasize the significance of delving into the sensory aspects of Taiwanese-style thick soup variations, ultimately enhancing our comprehension of its gastronomic encounters.

### 5.2. Limitations and Future Research Directions

In conclusion, the research results indicate the potential for future exploration in incorporating balsamic vinegar into Chinese cuisine, thereby expanding the spectrum of condiments used in Taiwanese-style thick soup beyond black vinegar. However, it is imperative to acknowledge the limitations of this study. 

Consumer preferences for condiments are inherently subjective, with individuals possessing distinct taste inclinations. While sensory attributes such as appearance, aroma, and taste contribute to consumer decision making, it is challenging to significantly modify these preferences based solely on sensory aspects. Furthermore, it is crucial to recognize that the participants in this study were predominantly Taiwanese and primarily composed of young adults (20–29 years old), which restricts the applicability of the findings to other cultural contexts and ethnic groups. Differences in individuals’ background information can lead to variations in food acceptance and willingness to make dietary adjustments [49]. Age disparities also influence the reception of novel and functional foods [48,50,51]. Hence, future research should strive to encompass individuals from diverse ethnic backgrounds to facilitate a more comprehensive discussion and evaluation.

For professionals and developers in the food industry, it is advisable to consider both the cultural context and consumers’ familiarity with specific condiments—a significant strength of this study. When introducing new products or condiments, it is pivotal to prioritize cultural exploration within the local market. During the initial stages of product development, emphasis should be placed on disseminating pertinent knowledge and creating awareness. This approach will heighten the potential for successful localization and foster the consumer acceptance of diverse ethnic foods or condiments during the international marketing process, along with being a pivotal factor in food choices.

## Figures and Tables

**Table 1:** Demographic profiles of the participants of this study.

Category	Subcategory	Frequency	Percentage (%)
Gender	Female	100	59.9
	Male	67	40.1
Age group	20 to 29 years old	87	52.0
	30 to 39 years old	40	24.0
	40 to 49 years old	21	12.6
	50 years old or above	19	11.4

**Table 2:** Taiwanese-thick-soup-making procedure.

Production Steps	Production Method
Step 1	Shred the material for production
Step 2	Boil a pot of water
Step 3	Add shredded bamboo shoots, shredded mushrooms, and shredded cabbage
Step 4	Add egg mixture and stir
Step 5	Seasoning and thickening
Step 6	Add cilantro and vinegar to finish

**Table 3:** Sensory evaluation questionnaire for soup and vinegar.

Sensory Visual	1. I think the color of the soup is very appropriate
	2. I think the luster of the soup is very appropriate
Sensory Olfactory	3. The soup smells very appropriate
	4. The vinegar smell of the soup is very appropriate
Sensory Taste	5. After the soup enters my mouth, I feel that the sour taste is very appropriate
	6. After the soup entered my mouth, I feel that thickness and smoothness were very appropriate
	7. After the soup enters my mouth, I feel that the umami taste is very appropriate
	8. After the soup entered my mouth, I feel that the sweetness was very appropriate
Sensory Overall	9. The overall vision, smell and taste of soup make me feel very enjoyable

**Table 4:** Grouping test table.

Group	Number	Descriptive Statistics		Variance Homogeneity Test		Independent Samples *t*-Test	
		M	SD	F	*p*	*t*	*p*
Traditional seasoning (black vinegar)	80	3.27	0.76	0.85	0.36	1.49	0.14
Innovative seasoning (balsamic vinegar)	87	3.45	0.81				

**Table 5:** The visual sensory evaluation test of traditional Taiwanese-style thick soup and innovative Taiwanese-style thick soup.

Item	Sensory Visual (Control Group)		Sensory Visual (Experimental Group)		*t*	*p*-Value
	M	SD	M	SD		
Traditional seasoning (black vinegar)	2.53	0.83	2.93	0.93	−2.98	0.00
Innovative seasoning (balsamic vinegar)	3.71	0.73	4.00	0.86	−2.31	0.02

**Table 6:** Olfactory sensory evaluation comparison of traditional Taiwanese-style thick soup and innovative Taiwanese-style thick soup.

Item	Sensory Olfactory (Control Group)		Sensory Olfactory (Experimental Group)		*t*	*p*-Value
	M	SD	M	SD		
Traditional seasoning (black vinegar)	3.04	0.91	3.36	0.75	−2.49	0.01
Innovative seasoning (balsamic vinegar)	4.10	0.88	4.47	0.70	−3.04	0.00

**Table 7:** Taste sensory evaluation comparison of traditional Taiwanese-style thick soup and innovative Taiwanese-style thick soup.

Item	Sensory Taste (Control Group)		Sensory Taste (Experimental Group)		*t*	*p*-Value
	M	SD	M	SD		
Traditional seasoning (black vinegar)	3.34	1.06	3.60	0.80	−1.81	0.07
Innovative seasoning (balsamic vinegar)	4.09	0.75	4.28	0.68	−1.71	0.09

**Table 8:** Overall sensory evaluation comparison of traditional Taiwanese-style thick soup and innovative Taiwanese-style thick soup.

Item	Sensory Overall (Control Group)		Sensory Overall (Experimental Group)		*t*	*p*-Value
	M	SD	M	SD		
Traditional seasoning (black vinegar)	3.10	0.88	3.54	0.94	−3.12	0.00
Innovative seasoning (balsamic vinegar)	3.88	0.88	4.20	0.85	−2.40	0.02

## Data Availability

Not applicable.
